# Perspective: Protocells and the Path to Minimal Life

**DOI:** 10.1007/s00239-024-10197-6

**Published:** 2024-09-04

**Authors:** David Deamer

**Affiliations:** Department of Biomolecular Engineering, University of California, Santa Cruz, CA USA

**Keywords:** Protocells, Self-assembly, Membranes, Polymerization, Encapsulation

## Abstract

The path to minimal life involves a series of stages that can be understood in terms of incremental, stepwise additions of complexity ranging from simple solutions of organic compounds to systems of encapsulated polymers capable of capturing nutrients and energy to grow and reproduce. This brief review will describe the initial stages that lead to populations of protocells capable of undergoing selection and evolution. The stages incorporate knowledge of chemical and physical properties of organic compounds, self-assembly of membranous compartments, non-enzymatic polymerization of amino acids and nucleotides followed by encapsulation of polymers to produce protocell populations. The results are based on laboratory simulations related to cyclic hydrothermal conditions on the prebiotic Earth. The final portion of the review looks ahead to what remains to be discovered about this process in order to understand the evolutionary path to minimal life.

## Introduction

What do we mean by minimal life? One way to address the question is to trace the initial steps from simplicity to complexity with one end of the complexity spectrum being the transition to protocell populations. A bottom-up approach will be used to describe the stages of increasing complexity that have been established by laboratory research and seem plausible in a prebiotic world. Some of these are spontaneous processes while others require a source of energy. This perspective will start with the conditions of a sterile prebiotic Earth and then incrementally add complexity until we reach the transition from protocells to minimal life when the system becomes sufficiently complex to grow by energy-dependent polymerization guided by genetic information.

The steps that will be considered include sources of organic compounds on the early Earth, self-assembly of membranous boundaries, non-enzymatic polymerization of amino acids and mononucleotides, and encapsulation of polymers to produce populations of protocells. An important caveat: Although the process is shown as steps, this is just a convenient way to put multiple processes into a logical order. Many of the steps proceed simultaneously as physical and chemical interactions occur in a mixture of thousands of organic and inorganic solutes present on the prebiotic Earth. The evidence presented here has led this author to propose that the most conducive environment for such a process is subaerial freshwater hot springs. Knowledgeable colleagues might choose a different site, but the end result will still incorporate a defined set of chemical reactants and physical processes that lead to complex systems of polymers capable of capturing energy and nutrients to grow and reproduce.

## Sources of Organic Compounds on the Early Earth

Research on minimal life begins with assumptions about what the Earth was like four billion years ago, and what organic compounds were present. The following assumptions will provide a background to guide the discussion here. The atmosphere in the early Archean was nitrogen (N2) mixed with smaller amounts of carbon dioxide and water vapor. The Earth was covered by a slightly acidic global ocean with ionic solutes such as NaCl, MgCl2, CaCl2 similar to those concentrations today. Volcanic land masses resembling Hawaii and Iceland emerged from the ocean and were supplied with freshwater distilled from the salty ocean and falling as rain. Two estimates based on the composition of certain minerals concluded that the global temperature was hot, ranging from 55 to 85 °C (Knauth and Lowe 2003; Robert and Chaussidon 2006) but a recent estimate taking into account the effects of impact ejecta proposed a more moderate 0 to 40 °C (Kadoya et al. 2020). These, of course, are estimates of global temperature ranges that do not take into account the higher temperatures of localized hydrothermal sites associated with volcanism.

A basic assumption is that reactive compounds like cyanide (Ferris and Hangan 1984; Pearce et al. 2022) and formaldehyde (Pinto et al. 1980; Masuda et al. 2021) were generated and became sufficiently concentrated to react. The reactions are well known because they have been extensively studied in laboratory simulations. They include amino acids as products of the Strecker synthesis, carbohydrates from the formose reaction, nucleobases from hydrogen cyanide reactions, and hydrocarbon derivatives such as fatty acids and alcohols from Fischer–Tropsch synthesis (Nooner et al. 1976; McCollom et al. 2010). Peters et al. (2023) reported that meteoritic iron could catalyze the reduction of carbon dioxide by hydrogen at elevated temperature and pressure, producing a variety of 1 and 2 carbon compounds (methanol, ethanol, and acetaldehyde) together with small amounts of alkanes. Sponer et al. (2016) have proposed that formamide is a plausible alternative to HCN for the prebiotic synthesis of nucleobases. It has also been suggested that hydrogen gas emitted by alkaline hydrothermal vents could reduce carbon dioxide to initiate a primitive version of metabolism (Martin et al. 2008).

**Fig. 1:**
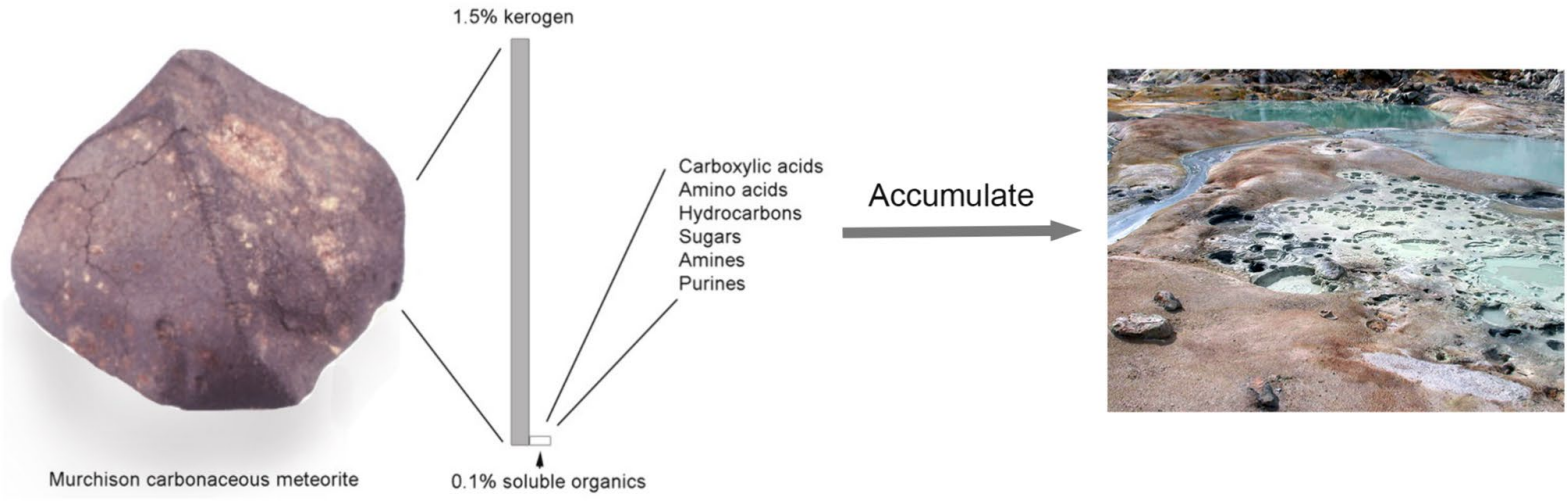
The Murchison meteorite contains most of the varieties of organic compounds that would be required for the assembly of protocells. The organic content of IDPs and carbonaceous meteorites would presumably accumulate and become concentrated in hydrothermal fields resembling Bumpass Hell on Mount Lassen National Park in California

Although it seems likely that geochemical reactions could have served as sources of organic compounds required for the origin of life, there is no evidence that such reactions did occur on the prebiotic Earth. There is direct evidence for the second potential source of organic compounds because the process is still happening today. Radioastronomy has identified 180 carbon compounds in the molecular clouds of dust and gas which give rise to stars, planets, and solar systems (Ehrenfreund and Sephton 2006). The organic compounds are present in icy films on microscopic dust particles composed of silicate minerals (Allamandola et al. 1998) and then preserved in comets and asteroids that accrete from the dust. The strongest evidence favoring delivery of extraterrestrial organics to the early Earth is their presence in interplanetary dust particles (IDP) collected in the upper atmosphere. They are also present in carbonaceous meteorites (Fig. 1) that contain thousands of organic compounds including amino acids, nucleobases, simple carbohydrates (Pizzarello 2006; Callahan et al. 2014), and monocarboxylic acids with hydrocarbon chains long enough to have amphiphilic properties. Chyba and Sagan (1992) calculated the amount of organic carbon delivered by IDPs and estimated a total of ~ 10^8^ kg carbon per year 4 billion years ago.

Is that sufficient for life to begin? The answer is uncertain, but an alternative has been suggested in which impacts of comets could have produced craters containing concentrated mixtures of their organic content (Clark and Kolb 2018). The authors referred to such craters as “Procreative Darwinian Ponds” and proposed that smaller icy comets could undergo atmospheric entry and impacts in such a way that their organic content would not be destroyed by extreme temperatures. The advantage of this scenario is that it bypasses the requirement for a concentrating process and immediately provides a mixture of organic compounds similar to that known to be present in icy comets.

Pearce et al. (2024) have proposed yet another solution. They begin with the assumption that very early in the Earth’s history as a planet the atmosphere resembled that of Titan in which an abundant haze of organic compounds dominated its composition. Laboratory simulations of such hazes with methane as a carbon source were run for 6 days, and the compositions of organic compounds synthesized were established by mass spectrometry. The remarkable result was that all five nucleobases present in nucleic acids had been synthesized as well as nine amino acids that compose proteins in living cells today. A computational model relating rates of synthesis and degradation was then used to estimate the maximum concentration of nucleobases in hydrothermal ponds, which ranged from 5 to 20 micromolar for adenine, guanine, and xanthine, up to 100 micromolar for uracil and thymine.

To summarize, although the source of reactive organic species remains uncertain, we can be reasonably confident that compounds resembling those in carbonaceous meteorites were available on the prebiotic Earth. For the purpose of this perspective, we will assume that both geochemical synthesis and extraterrestrial infall contributed to the suite of organic compounds. Most of these would be diluted in the global ocean and be unable to participate in chemical reactions leading to life’s origin, but those that fall onto subaerial land masses produced by volcanism would accumulate over time. This review will now focus on reactions that can occur when organic compounds become solutes in freshwater hydrothermal fields (Deamer et al. 2019; Damer and Deamer 2020). For a more detailed and comprehensive review of conditions on the early Earth, see Westall et al. (2023).

### In Order to React, the Organic Compounds Must be Concentrated

Organic substances falling on dry land are unable to participate in chemical reactions, so it is reasonable to assume that precipitation must flush them downhill as dilute aqueous solutions into hydrothermal fields associated with volcanism. Precipitation is distilled by evaporation from a salty global ocean, which means that freshwater with minimal ionic solutes would be available as a solvent for the organic compounds required for life’s origin. Sulfur dioxide (SO2) is a component of volcanic gas emissions along with CO2 and water vapor. When SO2 dissolves in water it becomes hydrated to sulfurous acid, which is a weak acid with a pKa of 1.85 that lowers the pH of typical volcanic hydrothermal fields to between 2 and 3.

Freshwater solutions of organics in subaerial hydrothermal fields have another significant feature. They can undergo continuous cycles of evaporation and rehydration at an elevated temperature. As the solutions evaporate, solutes become increasingly concentrated and finally form a film on mineral surfaces. Chemical reactions that cannot occur in dilute aqueous solutions become possible in concentrated solutions and in the organic films of potential reactants. Even if only small amounts of organic compounds are present in solution, it is possible that the chemical reactions required for life to begin occurred in thin films on mineral surfaces.

Chemists and biochemists prefer to use pure reagents in the laboratory and traces of contaminating impurities are avoided to the extent possible. The fact that dilute aqueous solutions on the prebiotic Earth were likely to contain thousands of different organic and inorganic compounds is daunting. How can specific reactions related to living systems occur in such messy conditions prior to life’s origin? A few reactants have physical properties that allow them to become spontaneously concentrated and purified, a common example being the self-assembly of amphiphilic compounds into monolayers, micelles, and bimolecular membranes, as will be described in the next section.

### Certain Molecules Spontaneously Assemble into Boundary Structures Required by Cellular Life

Soap bubbles are familiar to everyone from their experience as children. They are so common that their extraordinary properties are seldom appreciated. A general term describing organic compounds like soap is that they are amphiphilic: the nonpolar hydrocarbon chain is hydrophobic and the head group is hydrophilic. Certain amphiphilic compounds called lipids spontaneously assemble into lipid bilayers that provide the membranous boundaries of all living cells. In the absence of amphiphilic compounds cellular life could not exist.

A typical soap molecule consists of a hydrocarbon chain 12 to 18 carbons in length with a carboxylate group at one end. Soap molecules are also called fatty acids because they compose the triglycerides called fat. Understanding the physical properties of fatty acids is central to the origin of membranes required by cellular life. Imagine that a solution of soap in a bowl is set aside on a shelf and monitored. Hours can pass, and then days, but nothing happens because the solution is at chemical and physical equilibrium. Then imagine that a little air is blown into the solution through a straw. Even with this minimal input of energy, soap bubbles immediately form on the surface and may even float away into the air. How can this possibly happen?

**Fig. 2:**
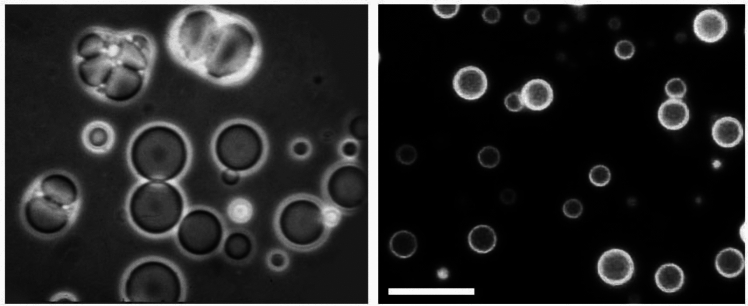
Monocarboxylic acids with chain ranging up to 12 carbons in length are present in the mixture of organic compounds in the Murchison meteorite (**A**). Even though they are more than 5 billion years old, they readily assemble into membranous vesicles. **B** The vesicles are fluorescent because polycyclic aromatic hydrocarbons are components of the membranes. Bar shows 20 µm. Credit: author

A soap bubble is a kind of inverted membrane in which the hydrocarbon tails of soap molecules project outward from each side of the membrane into the air and the hydrophilic head groups are directed inward, stabilized by their interaction with a thin film of water. In the bulk phase solution of soap, two other kinds of structures assemble at the nanoscopic and microscopic level. Soap molecules can exist in solution as individual molecules, but at a certain concentration they spontaneously form structures called micelles. A typical micelle is a spherical aggregate a few nanometers in diameter composed of several hundred molecules with their hydrocarbon chains directed into the interior and hydrophilic head groups interacting with water on the micellar surface. When the soap solution becomes further concentrated, the micelles begin to fuse into membranous bilayers that form microscopic compartments called vesicles or liposomes. Could this occur in the prebiotic environment? The answer is yes. The suite of organic compounds in carbonaceous meteorites include long chain monocarboxylic acids, and when these are extracted and exposed to water, they assemble into vesicles (Deamer 1985). The property of self-assembly has been preserved in the asteroid parent bodies even though the amphiphilic mixture is over five billion years old (Fig. 2).

An alternative to membranous compartments containing polymers should be mentioned here. A computational model referred to as GARD (graded autocatalysis replication domain) does not depend on genetic information in linear polymers (Segre et al. 2000). Instead, the microscopic structures resemble micelles and coacervates, and it is their composition that can presumably evolve and be inherited.

### Other Molecules are Monomers that Undergo Energy-Dependent Non-Enzymatic Polymerization

There are two experimental approaches to investigating prebiotic polymerization. The first is guided by the polymerization process of life today which is driven by chemical activation of monomers. For instance, protein synthesis of peptide bonds uses hydrolysis of the anhydride bond of ATP to synthesize aminoacyl adenylate, and a second enzyme-catalyzed reaction transfers the aminoacyl group to tRNA with the result that amino acids are attached to tRNA through a high energy aminoacyl bond. This makes it thermodynamically favorable to transfer activated amino acids to growing peptide strands on ribosomes. Nucleic acid synthesis also uses the chemical energy of nucleoside triphosphates to form ester bonds catalyzed by polymerase enzymes.

Activation of the monomers is essential for reactions in bulk phase water because of the thermodynamic barrier to spontaneous condensation reactions (Liu et al. 2020). Commercial synthesis of biopolymers also incorporates activated monomers such as carbodiimide derivatives for peptide bond synthesis and nucleoside phosphoramidites for ester bond synthesis of oligonucleotides. In the early 1980s Orgel found that imidazole esters of guanosine monophosphate could assemble on a polycytidylic acid template and polymerize into short oligonucleotides (See Orgel 2004 for a review.) Ferris et al. (1992) discovered that the imidazole ester of adenosine monophosphate can also polymerize on montmorillonite clay particles. More recently, Li et al. (2017) reported that 2-aminoimidazole activated nucleotides were superior activating agents.

The imidazole esters of mononucleotides led to significant advances in our understanding of non-enzymatic polymerization, but there is no consensus yet on whether prebiotic monomers could have been chemically activated on the early Earth. However, there is one prebiotic condition that bypasses the requirement for activation. Freshwater hydrothermal fields associated with volcanic activity undergo endless cycles of evaporation and refilling by precipitation. Well-known examples are the hot springs and geysers of Yellowstone National Park in Wyoming. Others include hydrothermal fields in Iceland, New Zealand, and the Kamchatka peninsula of eastern Russia.

The reason hydrothermal sites are significant for the origin of life is that polymerization of amino acids and nucleotides can be driven by condensation reactions that occur during wet-dry cycles. For instance, if a solution of amino acids is heated to dryness, a variety of chemical linkages spontaneously form including peptide bonds (Lahav et al. 1978). Similarly, if a solution of mononucleotides is dried at moderately elevated temperatures, ester bonds are synthesized that produce oligonucleotides (Rajamani et al. 2008; Guzman et al. 2014; da Silva et al. 2014), a process that has recently been confirmed by atomic force microscopy (Hassenkam and Deamer 2022). It is important to note that although nucleobases are present in the mixture of organic compounds associated with carbonaceous meteorites, nucleotides are not. It follows that nucleotides must have been synthesized by reactions occurring in the prebiotic environment. Powner et al. (2009) and Becker (2019) are exploring potential pathways by which this may have occurred.

### Degradation Reactions

Many spontaneous degradation reactions represent hurdles to be overcome when simulating non-enzymatic polymerization reactions in the laboratory, and presumably in the complex conditions of the prebiotic Earth. Examples include hydrolysis of ester and peptide bonds, deamination of cytosine, and depurination of purine nucleotides composing nucleic acids. Such damaging reactions occur continuously in life today and are repaired by specialized enzymes which would not be available in the prebiotic environment. In the absence of repair enzymes, how could polymers required for the origin of life resist hydrolytic breakdown?

The simplest answer is related to our understanding of chemical kinetics. If the rate of synthesis exceeds the rate of hydrolysis, polymers will accumulate. All life today depends on this difference in rates. Another factor is that assembled polymeric structures can have properties that make them more resistant to hydrolysis. For instance, the double stranded helix of RNA is more resistant to alkaline hydrolysis than a single strand, and a folded protein is more stable to hydrolysis than the denatured unfolded state. The increased stability of such structures allows them to accumulate in a steady state far from thermodynamic equilibrium.

For the purposes of this perspective, we will assume that polymers of amino acids and mononucleotides synthesized by wet-dry cycling accumulated in freshwater hydrothermal ponds. This did not occur just once. Instead, indefinite numbers of wet-dry cycles continued over thousands of years, each cycle producing new varieties of mixed polymers ranging from dimers to much longer polymers. The polymers undergo a continuous process of turnover and recycling driven by hydrolysis and synthesis.

### Random Sequence Polymers can be Encapsulated in Membranous Vesicles to Form Protocells

Microscopic membranous compartments became available to researchers after it was discovered that phospholipids spontaneously assemble into vesicles now called liposomes (Bangham and Horne 1964). These are composed of one or more lipid bilayers that represent barriers to the diffusion of ions such as sodium and potassium and polar molecules like amino acids and nucleotides. But were phospholipids available on the early Earth? Probably not. Phospholipids are composed of two fatty acids, a glycerol, a phosphate and headgroups such as choline, ethanolamine, or serine, and their synthesis requires multiple enzyme-catalyzed steps. However, simpler molecules like fatty acids can also assemble into vesicles (Hargreaves and Deamer 1978) and are often used as models of prebiotic membranous compartments.

Membrane permeability to solutes is an important consideration as we titrate the steps of complexity that result in the transition to minimal life. A simple way to intuitively understand the range of permeability is to compare how readily various solutes pass through lipid bilayer membranes. There are three properties of solutes that affect permeability: size, polarity, and ionic charge. The primary property of amphiphilic compounds affecting permeability of the membranes they compose is the length of hydrocarbon chains and the components of their mixtures (Paula et al. 1996). For instance, phospholipids with 14 to 18 carbons in their hydrocarbon chains readily assemble into stable liposomes, phospholipids with 12 carbon chains form fragile and relatively permeable bilayer membranes, while those with 10 carbon chains cannot form stable membranes.

Permeability is measured in terms of the permeability coefficient (*P*) which has units of centimeters per second. *P* is defined by the measured flux *J* of the solute per unit area of a membrane down a concentration gradient ∆C. For small molecules like water crossing a typical lipid bilayer membrane *P* ~ 10^–3^ cm/s, but as the size of solute molecules increases *P* decreases dramatically. For instance, for glycerol, composed of three carbons and three hydroxyl groups, *P* ~ 10^–6^ cm/s and for glucose with six carbons and six hydroxyls *P* ~ 10^–10^ cm/s. Glycerol and glucose are polar molecules, but the permeability coefficient of ionic solutes such as potassium ions is ~ 10^–12^ cm/s, nine orders of magnitude slower than water.

Concentration gradients of ions across membranes are essential to all life, and the very low permeability of membranes to ions allows gradients to be maintained after they are generated by active transport. The barrier provided by lipid bilayers is also important for the assembly of protocells, defined as lipid vesicles containing potentially functional polymers. Polymers synthesized by the process described earlier are very large molecules and will have multiple polar and ionic groups as components. Therefore, they are virtually impermeable to lipid bilayer membranes. But if that is the case, how can they become encapsulated?

**Fig. 3:**
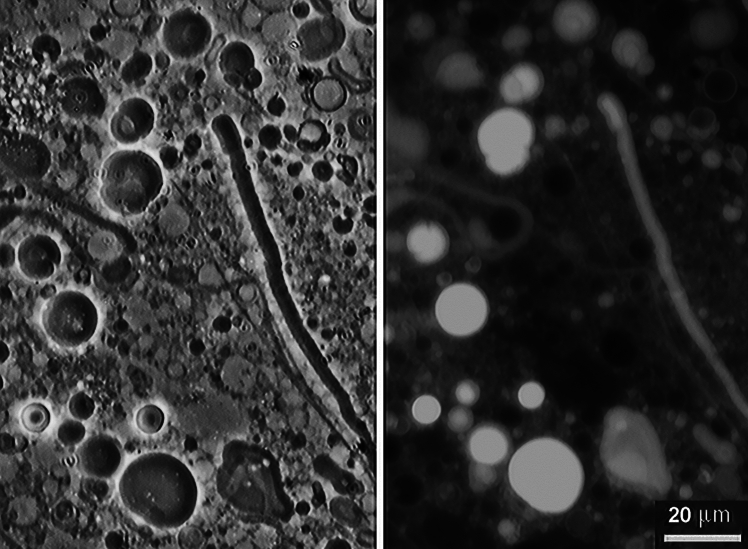
Protocell assembly. Dodecanoic acid in a 1:1 mol ratio with its monoglyceride were dispersed in water (10 mM) together with genomic DNA (1 mg/mL) that had been fragmented by sonication into short strands averaging around 600 nt in length. The mixture was dried on a microscope slide, then rehydrated with a dilute solution of acridine orange in water. The left image shows a phase micrograph of the preparation compared with a fluorescence image on the right. The encapsulated DNA is highly fluorescent because the acridine orange binds to the double helix. Credit: author

The answer to this question is surprisingly simple. The same wet-dry cycles that drive polymerization of monomers also promote encapsulation if lipids are present. If lipid vesicles are dried in the presence of polymers, the vesicles fuse into multilamellar structures with the polymers trapped between the layers of hydrophilic lipid head groups (Toppozini et al. 2013). When the multilamellar structures are rehydrated, lipid vesicles assemble again but this time contain encapsulated polymers. Figure 3 shows how easily polymers as large as DNA can be encapsulated by a single wet-dry cycle.

## Conclusion: From Protocells to Minimal Life

Protocells are defined as membranous compartments that contain mixtures of random sequence polymers. They are like microscopic test tubes, each different from all the rest and representing a kind of experiment in which their polymer composition and membranous compartments can undergo selection for properties that are related to living systems such as stability, permeability, catalytic activity, and replication. Most protocells would be initially inert and their components recycled because their polymers lack a sufficient number of the functions required for their survival. However, given that each function is selected because it increases the robustness of protocells to environmental stresses, the protocell population will evolve until a sufficient number of functions are incorporated for the transition to minimal life. It is conceivable that such a system could function using only RNA as the polymer, and for the purposes of this perspective they would be defined as minimal life: Membrane-encapsulated systems of polymers that capture energy and nutrients from the environment to grow by polymerization and then reproduce.

The phrase ‘minimal life’ is not intended to be synonymous with the last universal common ancestor (LUCA). Most would agree that LUCA would have all the attributes of microbial life today, incorporating a primitive metabolism, several hundred genes encoded in DNA, a genetic code, messenger RNA, transfer RNA and ribosomes that manufacture proteins. In contrast to protocells, LUCA was a product of evolution that began with minimal life followed by stepwise increases of biomolecular complexity over many millions of years.

Here are several questions that can guide future research with the goal of investigating the transition of protocells to minimal cellular life:

1. How did fragile protocells become stabilized to environmental stresses?
2. How were transmembrane pores inserted into protocell membranes that allowed the uptake of specific chemical nutrients?
3. How did a primitive metabolism become established in protocell populations?
4. How were random sequence polymers selected for catalytic activity?
5. How did protocells incorporate a cyclic process in which nucleic acids stored genetic information that could direct replication?
6. How were polymers selected for polymerase activity that allowed them to catalyze the synthesis and replication of genetic polynucleotides?

There are no simple answers yet to these fundamental questions, but significant progress has been made by exploring properties of ribozymes and encapsulated nucleic acids (Adamala and Szostak 2013). This progress has been summarized by Joyce and Szostak (2018); Joyce (2024) and Szostak (2017). Philipp Holliger and coworkers have discovered how to evolve ribozymes with polymerase activity (Wochner et al. 2011; Kristoffersen et al. 2022). Andes-Koback and Keating (2011) reported a system of lipid vesicles that could undergo a version of division into smaller daughter cells, and Zhu et al. (2012) described a similar process driven by photochemical redox potentials.

More recently, Toparlak et al. (2023) investigated a system of encapsulated polymers that fits the definition of protocells. It is interesting to consider this system in detail and then ask what further progress would be required to qualify as minimal life. The system incorporates vesicles composed of a single stranded ten-carbon cyclophospholipid (CPC10) mixed with dodecanol and dodecanoic acid in two different ratios: 2:3:5 and 6:3:1. The vesicles contain a short strand of RNA 18 nucleotides long paired with a fluorescently labeled primer 12 nucleotides long, leaving six nucleotides open at the end with the sequence CCCCAA. When nucleotides activated by 2-amino imidazole were provided to the system, they were able to pass through the vesicle membranes together with a magnesium ion-citric acid complex necessary for adding nucleotides to the open strand of the RNA. The 2-amino imidazole served as a leaving group so that an ester bond would extend the primer when an activated GMP formed a Watson–Crick base pair on the CCCCAA template. The system was incubated for 0, 2, 4, 7, 24, and 48 h. At 0 time only the fluorescent primer showed up in the gel, but as time went on the primer began to be lengthen by one nucleotide, then two and finally 3 nucleotides. If small vesicles composed of the lipids were added to the preparation, the lipid molecules were transferred spontaneously into the larger vesicles, causing them to grow by budding then finally divide into smaller vesicles that contained the template-primer duplex.

This system is one of the most technically advanced laboratory simulations of protocells and has several properties that fit the expectations of minimal life:

- Encapsulation of the system by a boundary membrane.
- The membrane is permeable to nutrients, and products accumulate inside the compartment.
- Template-directed growth occurs as activated monomers are added to the primer.
- If vesicles of amphiphilic compounds are added, they partition into the membrane and cause it to grow and then divide.

Now imagine that a system of such protocells happened to assemble spontaneously on the prebiotic Earth. What properties would be required for it to evolve into minimal life?

- The protocells must develop a primitive metabolism that generates chemical energy and uses nutrients diffusing through the membrane boundaries.
- The protocells will incorporate a catalyzed process by which polymers can be synthesized from activated monomers. If the monomers are ribonucleotides, the polymers will resemble RNA.
- In order to initiate replication, nucleic acids resembling RNA ribozymes must be able to use base sequences as a template to make copies of themselves.
- At some point regulatory controls using feedback begin to control rates of synthesis.
- To accommodate growth of the polymers, amphiphilic compounds can be added to the membranous boundaries.

The transition to the next level of complexity is even more demanding because a second polymer species is involved when activated amino acids begin to be assembled into proteins. The synthesis is guided by genetic information encoded in RNA and DNA, and the synthesis occurs in primitive versions of ribosomes. A variety of protein products begin to function including polymerases that catalyze the synthesis of nucleic acids. At some point in early evolution, protocells and minimal life emerged in natural conditions such as hydrothermal fields. Researchers are attempting to discover how that could happen, but the gaps in our understanding listed above are formidable and show how far we need to go to discover a process leading to minimal life. Furthermore, none of the laboratory simulations have faced the ultimate challenge of being demonstrated to function in natural conditions. We should be willing to test our assumptions because there is so much to learn by stepping out of the lab and performing reality checks in the field.
